# Adopting Learning Analytics to Inform Postgraduate Curriculum Design: Recommendations and Research Agenda

**DOI:** 10.1007/s10796-021-10183-z

**Published:** 2021-08-13

**Authors:** Denis Dennehy, Kieran Conboy, Jaganath Babu

**Affiliations:** National University of Ireland Galway, Galway, Ireland

**Keywords:** Learning analytics, Sentiment analysis, Business analytics, Curriculum design, Adoption

## Abstract

Understanding student sentiment plays a vital role in understanding the changes that could or should be made in curriculum design at university. Learning Analytics (LA) has shown potential for improving student learning experiences and supporting teacher inquiry. Yet, there is limited research that reports on the adoption and actual use of LA to support teacher inquiry. This four-year longitudinal study captures sentiment of postgraduate students at a university in Ireland, by integrating LA with the steps of teacher inquiry. This study makes three important contributions to teaching and learning literature. First, it reports on the use of LA to support teacher inquiry over four one-year cycles of a Master of Science in Business Analytics programme between 2016 and 2020. Second, it provides evidence-based recommendations on how to optimise LA to support teacher inquiry, with specific attention as to how these can improve the assimilation of LA into the curriculum design and delivery. Third, the paper concludes with a research agenda to help improve the adoption and integration of LA in the future.

## Introduction

The value of capturing student sentiment has received increasing attention by researchers in higher education (Knight et al., 2020; Linnenbrink-Garcia & Pekrun, 2011). Apart from obvious benefits to helping student well-being, understanding such sentiment is valuable to understanding the changes that could or should be made in curriculum design (Dunbar et al., 2014; Baxter-Magolda, 2003). Students’ sentiment is recognised as an integral part of student learning and a critical element in the learning process (Linnenbrink-Garcia & Pekrun, 2011; Henritius et al., 2019) as it is closely intertwined with students’ motivations and strategies for learning, self-regulation, performance and academic achievements (Mainhard et al., 2018; Mega et al., 2014; Pekrun et al., 2002). There are concerns, however, that research on student sentiment at university is fragmented (Mega et al., 2014). A focus on the perspectives of students is essential to the development of analytics related to their needs, rather than to the needs of institutions (Ferguson, 2012).

LA has emerged as an area with high potential for improving student learning experiences and curriculum design (Henritius et al., 2019; Ferguson, 2012). It belongs to a suite of ‘smart technologies’ (e.g., big data), also referred to as **‘**intelligent technologies’ that are increasingly being promoted as the solution to ‘smart education’ (Zhu et al., 2016) as their use focuses on how learning data can be utilised to improve teaching and learning (Mayer-Schönberger & Cukier 2013; Picciano 2012). LA involves the use of *“analytic techniques integrated with learning outcomes assessment to better understand student learning and more efficiently and meaningfully target instruction, curricula and support”* (Bach, 2010, p. 2). Integrating LA with teacher inquiry has been identified as critically important (Bos & Brand-Gruwel, 2016; Lockyer et al., 2013). Yet, there is a scarcity of research that examines the adoption of LA to support teacher inquiry (Dyckhoff et al., 2013; Mor et al., 2015; Sergis & Sampson, 2017). Moreover, educators may not always have the discretion to adopt work related technologies such as LA, as this decision is usually made at the organisation or departmental levels (Orlikowski, 1993; Fichman & Kemerer, 1997). We use the assimilation stages of innovation framework proposed by Gallivan (2001) as it acknowledges that adoption of technology is not always made by the individual (Cooper & Zmud, 1990).

Despite the increased attention that LA has received from researchers in recent years, “little research attention has been placed on providing recommendations to educators for translating the analysed data to actionable reflecting actions on their educational design and delivery” (Sergis & Sampson, 2017, p. 20). Further, LA has traditionally been applied to understand and optimise the learning process at module level, even though it can also be used to understand and optimise learning at the program level (Ochoa, 2016).

To address this gap in knowledge, the overarching aim of this study is to explore “*how adopting learning analytics can be used to understand students’ sentiment about their learning experience, and to use this understanding to inform teacher inquiry”.*

The focus of this study is important as there is a noticeable under representation of studies that directly engage with students in the shaping the curriculum design process curriculum and report the value of such engagements (Trowler & Trowler, 2010; Bovill, 2013; Campbell et al., 2007). We also provide recommendations, which is important because the process of obtaining actionable insights for curriculum design is generally considered to be a time-consuming activity for educators (Sergis & Sampson, 2017; Marsh & Farrell, 2014; Mor et al., 2015).

In the context of this study, we adopt the view that the student voice is about actively involving students in evaluating and redesigning curriculum (Bovill et al., 2011; Bovill, 2013; Trowler & Trowler, 2010) as it has a unique perspective on teaching and learning, and therefore, it warrants the attention and response of educators (Rudduck, 2007; Fielding, 2001; Hattie, 2008). Further, although the student voice has increasingly gained prominence in higher education (Campbell et al., 2007), the focus has primarily been on quality assurance with less attention given to active student involvement (Seale, 2009).

The remainder of this paper is structured as follows. First, the theoretical background to LA and teacher inquiry is presented. Next, the research method and background to the case studied is presented. Then, the findings and analysis are presented. Followed by discussion, recommendations, implications, and a research agenda. The paper ends with a conclusion.

## Theoretical Background

### Overview of Analytics

**Fig. 1:**
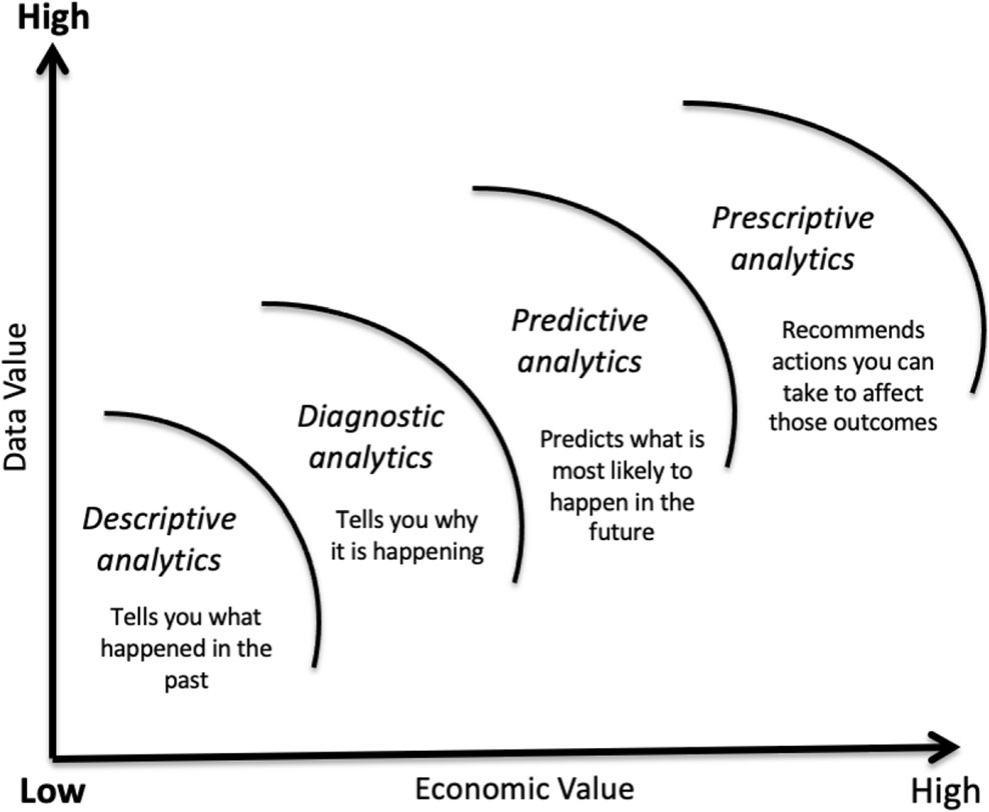
Types of analytics (Dennehy, 2020)

The term ‘analytics’ is interpreted differently across university stakeholders (Roden et al., 2017), be that across different academics, different academic departments and different business units. Analytics are categorised as descriptive, diagnostic, predictive, and prescriptive (see Fig. 1). In a broader context, analytics falls under the umbrella of ‘business analytics, a holistic approach that uses various technologies, methodologies, and applications to manage, process and analyse data that can lead to actionable insights and enable organisations to predict and respond to change. Business analytics have received increased attention from academics and practitioners to generate and use data for operational and strategic purposes to deliver business value (Chatterjee et al., 2021; Gupta et al., 2020; Wamba et al., 2015).

Understanding different analytics types will also inform the LA initiative, and specifically how the data will be modelled. As the context of this study is teaching and learning, the remainder of this section discusses the role of both academic and LA.

### Academic and lEarning Analytics

Academic analytics refers to the use of analytics within academic settings and may be applied at the level of the institution, the department, or the learner, depending on the goals and objectives of the analysis (Van Barneveld et al., 2012; Dunbar et al., 2014). In the context of this study it is used to transform teaching, learning, assessment, and curriculum design (Siemens & Long, 2011).

Academic analytics consist of two types of applied analytics called ‘institutional analytics’ and ‘learning analytics’ (Dunbar et al., 2014). *Institutional analytics* is generally used to understand factors that relate to running the business of the higher education institution, such as predicting student success and retention rates (Oblinger, 2012). *Learning analytics* focuses specifically on students and their learning behaviours (van Barneveld et al., 2012; Siemens & Long, 2011). LA was defined in 2011 by the Society for Learning Analytics Research at the 1st International Conference on Learning Analytics and Knowledge as *“the measurement, collection, analysis and reporting of data about learners and their contexts, for purposes of understanding and optimising learning and the environments in which it occurs”* (Ferguson, 2012). This definition includes techniques such as predictive modeling, building learner profiles, personalised and adaptive learning, optimising learner success, early interventions, social network analysis, concept analysis, and sentiment analysis. While there is no generally accepted definition of LA, it can play a critical role in understanding student learning and curriculum design, by supporting evidence-based practices derived from relevant measures of learning processes, outcomes, and activities (Mangaroska & Giannakos, 2018). Siemens and Long (2011) outline the differences between academic and LA (see Table 1).

**Table 1:** Differences between academic and learning analytics

Type	Level or object of analysis	Beneficiaries
Academic analytics	Institutional: learner profiles, performance of academics, knowledge flow	Administrators, funders, marketing
	Regional (state/provincial): comparisons between systems	Funders, administrators
	National and international	National governments, education authorities
Learning analytics	Course-level: social networks, sentiments, discourse analysis	Learners, faculty
	Departmental: predictive modeling, patterns of success/ failure	Learners, faculty

LA holds the potential to (i) detect and explain unexpected learning behaviours and misplaced efforts, (ii) identify successful learning behaviours and patterns, and (iii), introduce appropriate design interventions (Siemens & Long, 2011; Mangaroska & Giannakos, 2018). The link between LA and teacher inquiry is based on the premise that comprehensive data capturing and analysis is conducted at various levels (i.e. module, programme) to inform and influence the learning experience, the design process (or its ensuing refinement) and the community of curriculum designers (Hernández-Leo et al., 2019). There are several sources of information that can be used to analyse a program curriculum, with surveys about students’ perceptions and sentiments being the most popular tool in curricula analysis (Ochoa, 2016).

### Synergies Between Teacher Inquiry and Learning Analytics

Teacher inquiring is defined as a cyclical process in which “*teachers identify questions for investigation in their practice and then design a process for collecting evidence about student learning that informs their subsequent educational designs*” (Avramides et al., 2015, p. 249–250). Teacher inquiry is a process that can guide reflection and enhancement to curriculum design and delivery in a systematic and evidence-based approach (Dana & Yendol-Hoppey, 2014). In essence, teacher inquiry is a form of ‘action research’ as educators are in a position to determine questions to critically evaluate the design and delivery of their curriculum and choose appropriate data collection techniques (i.e., learning analytics) to answer these specific questions (Feldman et al., 2018; Sergis & Sampson, 2017). There are a number of generic steps involved in teacher inquiry which are listed in Table 2 and mapped to LA (Sergis & Sampson, 2017; Timperley et al., 2010; Hansen & Wasson, 2016). To the best of our knowledge, this is the first study to align learning analytics, with teacher inquiry.

**Table 2:** Mapping learning analytics with the steps of teacher inquiry

Step	Teacher inquiry cycle	Description (Sergis & Sampson, 2017; Hansen & Wasson, 2016; Timperley et al., 2010)	How LA can contribute to teacher inquiry	Literature sources
1	Problem identification	Identification of a specific aspect of educational design (i.e., module, programme) and/or delivery to be evaluated in order to improve it	Can be used to measure, collect, analyse and report on students’ learning experience and the context of their learning	(Ferguson, 2012, Papamitsiou & Economides, 2014)
2	Develop enquiry questions	Specific questions, data to be collected, and the method for data collection is established	Can be used to identify specific problems related to the module/ programme	(Mor et al., 2015, Sergis & Sampson, 2017)
3	Educational design	Formulation of educational design in which the teacher will deliver in order to implement their inquiry	Can be used to improve the learning experience for individual learners or groups of learners by modelling the data to inform educational design	(Ferguson, 2012, Papamitsiou & Economides, 2014, Bakharia et al., 2016)
4	Deliver educational design and collect data	Delivering the educational design to the learners and collects the educational data using the collection method	Can be used to collect relevant, high quality educational data that have been defined to answer their inquiry question	(Sergis & Sampson, 2017, Papamitsiou & Economides, 2014)
5	Analyse educational data	Data is analysed in order to elicit insights to answer the inquiry questions	Can be used to analyse and report on the collected data and facilitate sense-making and decision-making	(Sergis & Sampson, 2017, Papamitsiou & Economides, 2014)
6	Reflect on data	The analysed data are used in order to answer the defined inquiry question and revise the practice in which the educational design and/or delivery is practiced	Can be used as an evidence-based approach to guide reflection and actionable insights	(Dana & Yendol-Hoppey, 2014, Bakharia et al., 2016, Greller et al., 2014)

LA can also provide support for educators to reflect on and improve curriculum design and delivery through the evidence-based insights generated by LA (Bakharia et al., 2016; Sergis & Sampson, 2017; Greller et al., 2014). Therefore, LA supports the concept of teacher inquiry (Mor et al., 2015) and can be linked to the teacher inquiry cycle (Sergis & Sampson, 2017).

Despite the critical importance of integrating LA with curriculum design (Bakharia et al., 2016, Lockyer et al., 2013), there is limited research that reports on the actual use of LA to support curriculum design (Bakharia et al., 2016; Dyckhoff et al., 2013; Sergis & Sampson, 2017). Most concerning is that LA are increasingly being implemented in different educational settings, often without the guidance of a research base (Siemens, 2012). In addition, there is very little research on how to analyse the learning process at the program level in order to guide the design or redesign of a curricular program. Research does suggest, however, that educators may lack the skills and knowledge to formulate questions and identify solutions (Olah et al., 2010; Means et al., 2011) or they may not always know how to make sense of the data in order to inform curriculum redesign (Olah et al., 2010; Heritage et al., 2009; Young & Kim, 2010).

While many LA studies identify patterns in students’ learning behaviour, which are then related to academic performance, understanding of the pedagogical context that influences student activities is lacking (Lockyer et al., 2013; Gasevic et al., 2016). A related issue is the need to use the ‘actionable insights’ generated from the use of LA to make appropriate design interventions to improve learning (Clow, 2013; Campbell et al., 2007). Most concerning is that LA, an interdisciplinary field that adopts methods and frameworks from other disciplines, lacks a consolidated model to systematise how those disciplines are merged together (Gašević et al., 2017; Mangaroska & Giannakos, 2018).

### Assimilation Theory as a Means to Examine the Adoption of Learning Analytics

This study draws on innovation assimilation theory. Assimilation is defined by Meyer and Goes (1988, p. 897) as “an organisational process that (i) is set in motion when individual organisation members first hear of an innovations development; (ii) can lead to the acquisition of the innovation; and (iii) sometimes comes to fruition in the innovation’s full acceptance, utilisation, and institutionalisation”. We apply the assimilation framework for innovation adoption proposed by Gallivan’s (2001) who in turn was been heavily influenced by earlier work of Cooper and Zmud (1990). The framework has been used to study the diffusion and assimilation of information technology innovation (Fichman, 2000), software process innovations (Fichman & Kemerer, 1997), e-business (Zhu et al., 2006), and enterprise information systems (Liang et al., 2007; Saraf et al., 2013).

While several researchers have proposed various frameworks describing the technology implementation process in organisations, Gallivan’s framework is one of the most cited frameworks (Weible & Hess, 2018). In recent years it has been used to study contemporary technologies such as big data (Bharati & Chaudhury, 2019; Weible & Hess, 2018), cloud (Ooi et al., 2018), social media (Cao et al., 2018), and contemporary processes such as agile (Wang et al., 2012), and Kanban (Ahmad et al., 2018). The six assimilation stages of innovation are adapted for this study and are therefore restated in the context of LA rather than technology generally. These adapted definitions are listed and explained in Table 3.

**Table 3:** Stages of assimilation (adapted from Gallivan, 2001)

Assimilation phase	Explanation
Initiation	A match is found between the LA technology and its application in the organisation.
Adoption	A decision is reached to invest resources to accommodate the implementation of learning analytics.
Adaptation	The LA technology is developed, implemented and maintained, and members are trained to use the analytics.
Acceptance	Members are persuaded to commit to using the LA technology.
Routinisation	Usage of the LA technology is encouraged as a normal activity in the organisation.
Infusion	The LA technology is used in a comprehensive and sophisticated manner which is determined by three different facets of infusion, namely: • Extensive use: using more features of the technology. • Integrative use: using the technology to create now workflow linkages among tasks. • Emergent use: using the technology to perform tasks not envisaged in its initial design.

A strength of this framework is that is acknowledges the realities of adoption within organisations, particularly when adoption decisions are made at the organisation, departmental, or workgroup levels, rather than at the individual level (Orlikowski, 1993; Fichman & Kemerer, 1997).

## Research Method

### Background to the Case Studied

The research described in this paper follows the principles of a case study method (Yin, 2009). The context of the case is a one-year fulltime master’s programme in business analytics at a university in Ireland. The specific case was purposefully chosen because, (i) monitoring student sentiment was critical as the programme underwent significant growth each year, (ii) ensuring the programme was designed for inclusive teaching as the student population was diverse, and (iii) the programme director was keen that students had a positive student experience.

#### Background to the Case

The Master of Science (Business Analytics) is designed as a specialist programme, which assists students to blend their existing talents with the analytical skills and business knowledge needed to use and manage big data and business analytics in knowledge-based companies. The programme is aligned with *Ireland’s National Skills Strategy 2025* by placing a strong focus on providing skills development opportunities that are relevant to the needs of learners, society and the economy.

#### Programme learning outcomes

The learning outcomes are intended to equip students with the required industry-standard skills and knowledge: (A) understand and be able to use specific IT which is used in developing business analytics. (B) analyse and solve business problems using applied data analytics. (C) understand and apply techniques for managing IT in organisations. (D) identify, analyse and solve applied problems in individual and team-based settings. (E) apply effective data-driven decision-making to global business and social problems.

#### Programme Outline

The programme consists of 90 ECTS (European Credit Transfer and Accumulation System). Modules (see Table 4) are worth 5 ECTS, with the exception MS5103 Business Analytics Project (30 ECTS) and Business Analytics with third party software (10 ECTS). The programme commences in September and consists of three terms; September to Decembers (Term 1), January to April (Term 2), and April to August (Term 3).

**Table 4:** Modules offered for 2020-21 academic year

Term 1 (30 ECTS)	Term 2 (30ECTS)	Term 3 (30 ECTS)
• Business Modelling & Analytics	• Data Science & Big Data Analytics	• Business Analytics Project
• Database Systems	• Applied Customer Analytics	
• Business Applications Programming	• IS Security & Ethics	
• Decision Theory & Analysis	• Enterprise Systems	
• Statistical Techniques for Business Analytics	• Advanced Programming for Business Analytics	
• Systems Development & Project Management (Elective)	• IS Strategy & Innovation	
• Strategic Management (Elective) • Business Analytics with Third Party Software (Elective)		

#### Programme Reputation

The MSc (Business Analytics) programme is the largest of its kind in Ireland and is only one of two such programmes in Ireland that qualified to be ranked by Quacquarelli Symonds (QS) rankings in 2020 and 2021. In 2020, the programme was ranked No.1 in the world for ‘value for money’ and in 2021 it ranked in the top 43 % in Europe for ‘alumni outcomes’ and ‘thought leadership’. The programme was also awarded the *Dean’s Award for Inclusive Teaching and Learning (Team Award)* in 2019. These endorsements, coupled with an excellent team of academics and administrators, regular engagement with students and alumni, and sharing of student events, awards, and First Destinations reports on social media are possible reasons for the continued growth of the programme (see Table 5).

**Table 5:** Number of applications and enrolments between 2015 and 2019

	2015-16	2016-17	2017-18	2018-19
Applications	89	185	469	675
Enrolments	16	36	57	99

#### Student Profile

Students from Ireland, India, UK, France, Pakistan, Nigeria, Greece, Brazil, China, USA, Ghana, Germany, Mexico, Indonesia, and Malaysia are largely represented on the programme each year. Students present with a range of industry experience (e.g., 1–8 years) and their academic background is varied (e.g., engineering, information systems, statistics, economics, sports, arts, business).

### Data Collection and Analysis

**Fig. 2:**
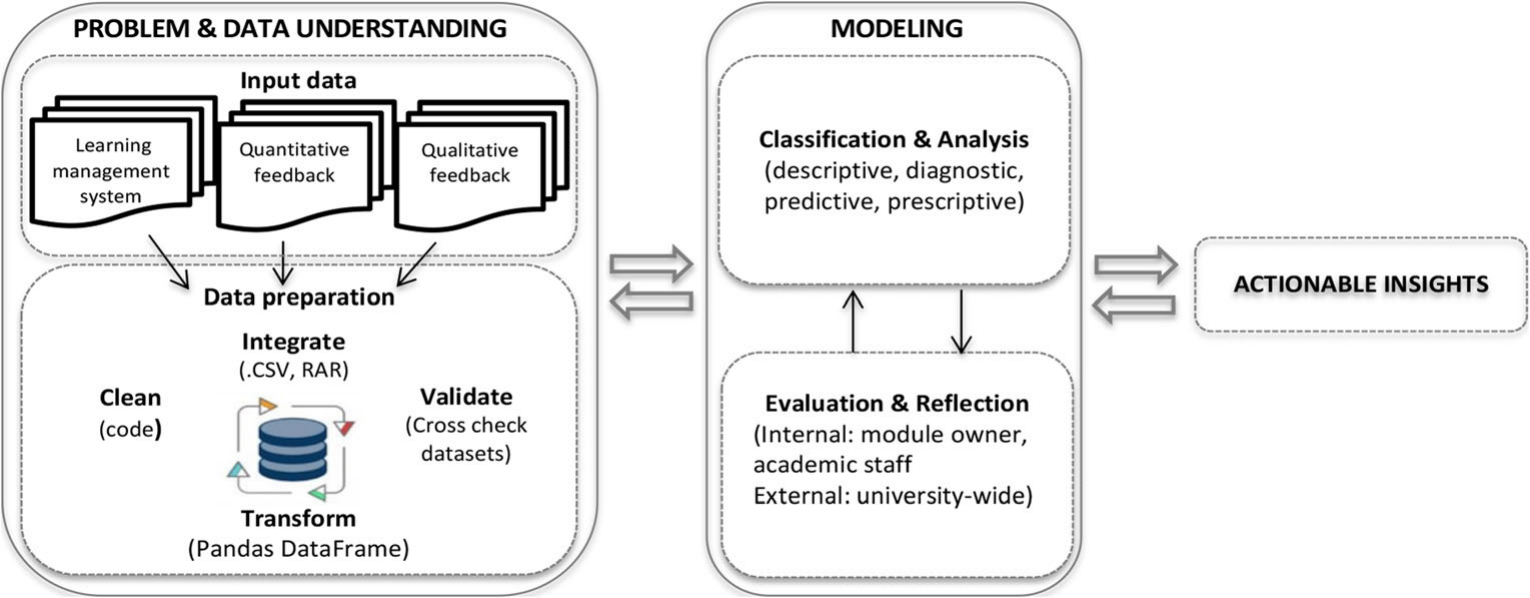
Learning analytics-based curriculum design framework

To address the concerns mentioned previously, we propose a LA-based curriculum design framework (see Fig. 2). The proposed framework is an adaptation of Cross Industry Standard Process for Data Mining (CRISP-DM), an industry standard methodology that prescribes a set of guidelines to guide the efficient extraction of information from data. The CRISP-DM methodology consists of six cyclical steps, namely (i) Business Understanding, (ii) Data Understanding, (iii) Data Preparation, (iv) Modeling, (v) Evaluation, and (vi) Deployment. We adapt this process methodology to suit the context of our research but do not exclude any of the six phases of CRISP-DM, instead, it merges them into three inter-related activities, namely, (i) Problem and Data Understanding, (ii) Modeling (i.e., classification, evaluation, and reflection) and (iv) Actionable Insights. Each of these phases are discussed below.

#### Problem and Data Understanding

This phase involves firstly understanding the problem in context and align the objective of the LA initiative with this problem. It is secondly about understanding what data sources are to be analysed to achieve this objective. In the context of teacher inquiry, input data consists of learning management system, quantitative data (i.e., surveys), and qualitative data (i.e., interviews, focus groups). Data preparation includes determining what data points to include in the dataset, extracting and cleaning the data.

#### Modeling

This phase comprises of data classification, type of analysis (i.e., descriptive, diagnostic, predictive, prescriptive), internal and/or evaluation (i.e. instructor, discipline). Evaluation and reflection of the emerging model and findings occur simultaneously, ideally in a collaborative team environment to ensure a shared understanding and shared commitment of the solution.

#### Actionable Insights

The emerging findings and actionable insights are then applied to the curriculum design problem identified and lessons learned shared with colleagues within the department and wider university setting.

The proposed framework is important, as curriculum design is a “methodology that educators use and communicate with each other to make informed decisions in designing learning activities and interventions with effective use of resources and technologies” (Conole, 2012, p. 121). It must also be conceptualised before it can be utilised as a process that leads to explicit design interventions and outputs.

### Instantiation of the Analytics-based Curriculum Design Framework

This section describes an instantiation of the proposed LA-based curriculum design framework that was previously discussed.

#### Problem and Data Understanding

In this phase, problem understanding focused on the context, aim and curriculum design problem in order to align with the LA initiative, and data understanding provided understanding of the data sources to be analysed. Primary input data that informed curriculum design comprised of (i) module data (i.e., 15 modules per year), (ii) programme reviews i.e., 1 per year), and (iii) interview data. The questions used for both the module and programme reviews are listed in Appendix Table 9 and were informed by constructive alignment and integrative learning literature (e.g. Biggs, 1996, 1999; Hounsell & Hounsell, 2007). In order to align with international accreditation bodies, student feedback questions for all modules delivered throughout the business school were standardised in 2016. These surveys are administered independently from the module owner (i.e., educator). Secondary data that informed curriculum design includes feedback from external examiners, accreditation bodies, and observations of similar programme offerings that are ranked by QS Rankings.

Qualitative data: To gain a rich understanding of the students’ learning context, interviews and observations were used as sources of evidence, as these techniques are particularly suited for increased immersion within the broader context of the case being studied (Yin, 2009; Stake, 2000). This data was collected throughout the academic years in the form of informal interviews with students and class representatives. Staff responsible for the design and delivery of the modules provided insight to the rational for the current pedagogical design, which enabled the researchers to unearth challenges associated with teaching and learning related to this programme.

#### Data Preparation

This phase included deciding what needed to be included in the dataset, cleaning the data and all other activities that needed to be done to process data which served as an input to the modeling tool in the next step. Data extraction and integration using Python scripts whereby messages were converted from RAR file format into .CSV file format. Text was then converted into Pandas DataFrame format for compatibility purposes with the sentiment analysis algorithm. Sentiment analysis refers to a sub-field of natural language processing (NLP) in computer science (Liu, 2010). Commonly, word dictionaries with pre-classified sentiments by linguists are used to determine sentiments in an automated manner using word counts (Liu, 2010; Tausczik and Pennebaker, 2010). Sentiment classification models can also be developed using state-of-the-art machine learning methods based on labelled datasets (Zhang et al., 2018). Sentiment analysis is the task of identifying positive and negative opinions, evaluations, gestures, and cultural meanings organised around a relationship to a social object, usually another person or group (Gordon, 2017; Wilson et al., 2005; Jongeling et al., 2015).

In this study, we use a different approach that is not based on textual analysis. While this is a viable method to assess qualitative feedback, we found that the amount of text per answer and the frequency in terms of number of students who replied to open-ended questions was not sufficient to deem this analysis credible by itself. We derive sentiments based on the replies to the Likert type questions. In this study, the five scale options of the Likert questions were rated between − 1 and + 1; disagree or strongly disagree rated as -1 (negative), neither agree nor disagree rated as 0 (neutral) and agree or strongly agree rated as + 1 (positive). For example, if there are 3 respondents for a given Likert question and the responses are, ‘*Strongly Disagree*’, ‘*Agree*’, and ‘*Strongly Agree*’, the aggregate score for that question is + 1.

#### Modeling

Essentially, this phase performed sentiment analysis across three consecutive academic years, namely, 2016-17, 2017-18, 2018-19, and 2019-20. To ensure high response rates, all responses were anonymised. The response rate for each end of year programme review was 72 % (2016-17), 96 % (2017-18), 70 % (2018-19), and 66 % (2019-20). As the response rate varied across the academic years, a number of analytical techniques were applied to calculate an overall rating scale of 0 to 5. Zero being the lowest overall score the programme could receive and five been the highest rating. To calculate the average of all the scores, we added individual scores for each of the 10 Likert questions (e.g., Q1 + Q2 + Q3 + Q4 + Q7 + Q8 + Q9 + Q11 + Q12 + Q13) and divided it by 10. The results of the data were then represented using Tableau, an industry standard analytical software tool that is used for interactive data visualisation.

#### Evaluation & Reflection

In this phase, the model, data, and emerging findings were analysed in relation to the problem and data understanding (e.g., disconnect between module and programme learning outcomes). This involved meeting with staff, students, and the research team. This iterative process ensured that the emerging findings led to ‘actionable insights’ that informed the curriculum design of the programme.

## Findings and Analysis

The findings and analysis presented in this section are intended to provide insight of how student sentiment and involvement influenced curriculum redesign rather than compare staff.

**Fig. 3:**
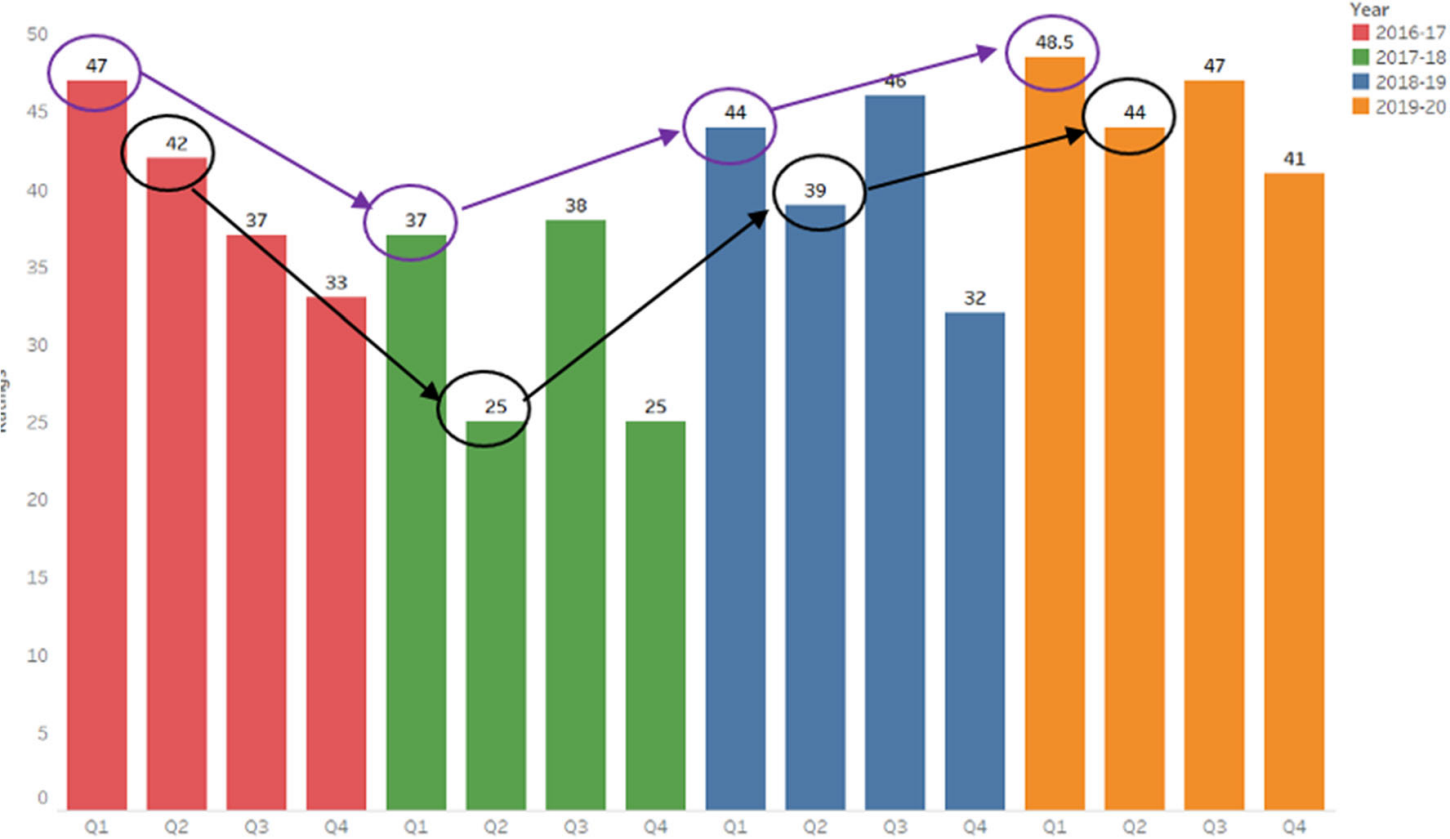
Sentiment of learning outcomes and assessment

The 2016-17 end of year programme review was the starting point of our empirical analysis as (i) this was the first programme review conducted since the programme commenced in 2015, (ii) the programme review was conducted by the incoming and newly appointed programme director, and (iii) this dataset provided a baseline from which to compare student sentiment in subsequent academic years. First, we were keen to understand if students were aware of the learning outcomes of the programme (Q1), if the programme delivered the expected learning outcomes (Q2), if the assessment and examination requirements were clearly communicated (Q3), and if the modules on the programme were linked effectively (Q4). The sentiment for each of these metrics is presented in Fig. 3. There was concern about a disconnect between the stated (see purple circles in Fig. 3) and realised learning outcomes and assessment (see black circles in Fig. 3).

To gain deeper insight of the sentiment ratings identified from the baseline survey, informal interviews with students and monthly meetings with the class representatives were conducted during the following academic years. Engagement with students was necessary in order to distinguish if there was a recurring pattern relating to curriculum design issues or if the issue was unique to the 2016-17 cohort of students. Engagement with students revealed that the majority of students did not distinguish between programme and module learning outcomes (Q1, Q2) and many students acknowledged that they did not know the programme learning outcomes or where to find them.

**Fig. 4:**
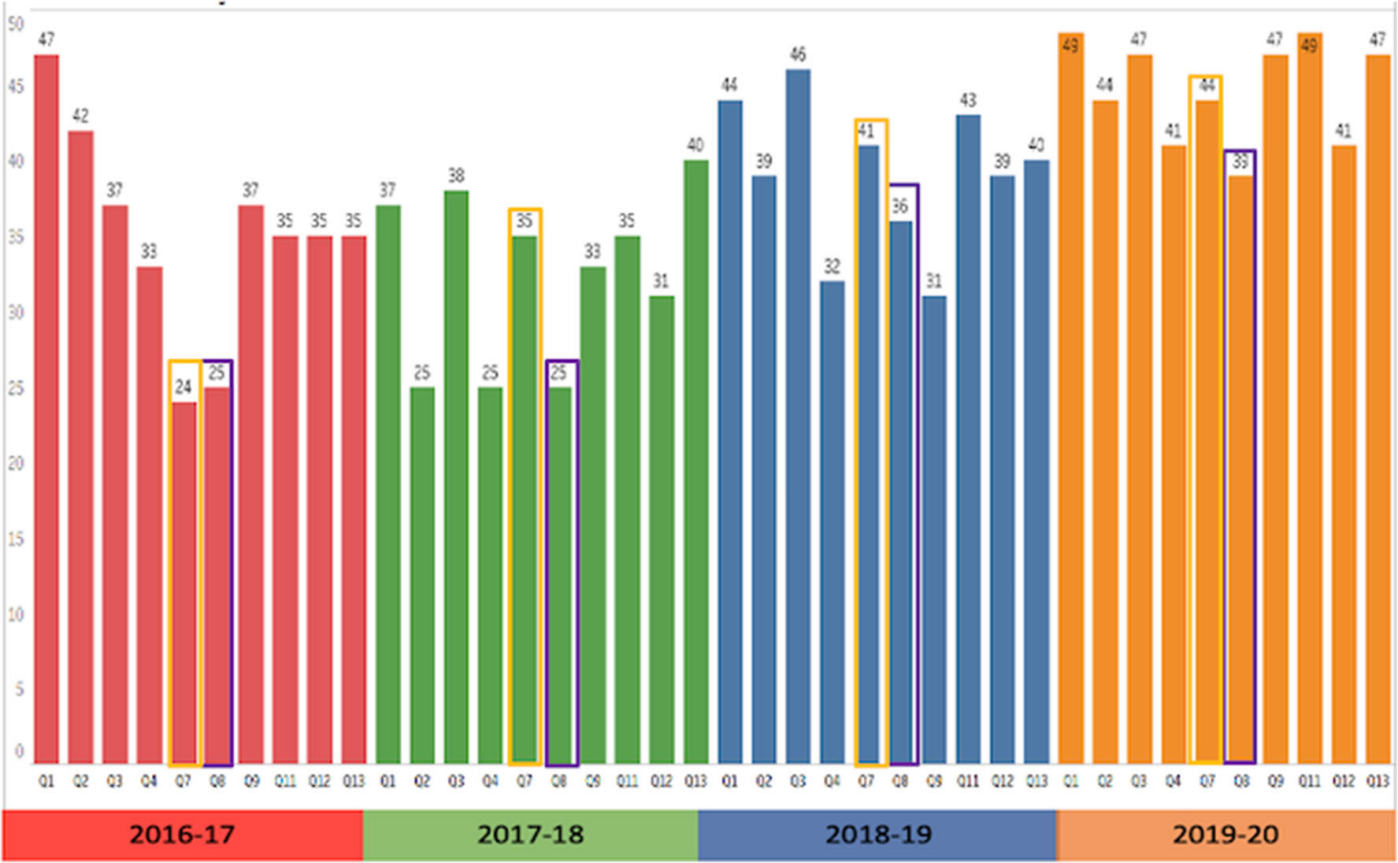
Curriculum design and delivery rating

A number of initiatives were implemented by the programme director and staff at the business school that has since positively increased student sentiment for the 2019-20 academic year (see Fig. 4 below). These included (i) designing a standard template for module descriptions with no more than five learning outcomes linked to a module, (ii) learning outcomes were based on Bloom’s taxonomy, (iii) learning outcomes of the programme and module descriptions with the associated learning outcomes were made available on the college website for current and potential students to review, and (iv) the programme learning outcomes were incorporated into the programme orientation and their relevance discussed with incoming students.

There was a concern that students (2016-17) did not find the programme intellectually stimulating (Q7). See the yellow squares in Fig. 4 for a comparison of each year. Students also reported that they did not receive helpful and/or timely feedback during the programme (Q8). This was surprising considering sentiment remained the same (25 out of 50 points) for the subsequent academic year (2017-18). However, interviews with students indicated that students were unable to identify when educators were providing ‘formative’ assessment compared to ‘summative’ feedback. This was concerning because incorrect assumptions about assessment do more damage by ‘misaligning’ teaching than any other single factor (Biggs, 2003). Staff now explicitly inform students when they are providing formative assessment and this had a considerable impact (rating of 39 out of 50) on student sentiment in the 2019-20 academic year, even though the class size had increased (see purple squares in Fig. 4).

Interviews (see Table 6) with students revealed that many students struggled to grasp ‘threshold concepts’ (Nicola-Richmond et al., 2018; Cousin, 2006; Meyer & Land, 2005) and/or were unable to apply the transferable skills obtained from a module to other modules (i.e. major project). Threshold concepts are important because business analytics is also a profession (cf. Land et al., 2018) that rely on evidence-based thinking and practices, that involves, key threshold concepts (e.g., digital literacy, appropriate use of business analytics terminology, critical appraisal of business analytics techniques and practices, and problem solving (technical, people, process).

**Table 6:** Sample of student feedback

Interviewee	Quote
Student 1	“The module turned out to be more of a reading exercise and just keep doing tasks as told like a robot and not grasping concepts and improvising data according to our needs”.
Student 2	“I thought it would be kind of helpful to be explained a little bit about why we were given the module. It would have been helpful to learn about how our experience with this module would benefit us, because I am not sure when this would fit in with my future career”.
Student 3	“Although I know much more than I used to about this subject, it could be much better. The fashion in which the module was delivered was very much passive and should not have involved more active components. The theory part of the tutorial covered so many aspects that it became very difficult to digest all of the information in one go. Moreover, it primarily focused on telling things rather than explaining or developing my understanding”.
Student 4	“I found the theory long at times and it was easy to lose focus. I don’t think I’m suited to this learning style, instead I find demonstrations or video tutorials easier and more enjoyable to follow. The long readings left me frustrated and I would personally benefit more from video tutorials”.
Student 5	“The sheer amount of ‘lecture materials’ that accompanied the course was substantial and, to say the least, I struggled with getting through them”.
Student 6	“As much as I was interested in mastering this subject, I am pretty much disappointed by the way the tutorials were carried out. Visual explanations help me to maintain my interest in the subject and acquire greater knowledge rather than going through loads of theoretical explanations”.

**Fig. 5:**
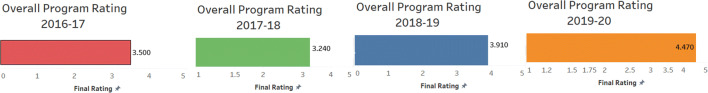
Programme rating for each academic year

The experiences listed above are reflected in Fig. 5, which presents sentiment trend over the four academic years. To help students ‘connect the dots’ between modules and to get a grasp of threshold concepts, the programme director initiated and supported a number of curriculum design changes. These included, inviting industry experts to share their experiences of using analytical tools and techniques in their respective industry, setting up a student-led business analytics society, and appointing an Honorary Professorship of the programme to a Chief Innovation Officer (CIO) of an analytics-centric multinational that has a presence in the country. The CIO visits the university to deliver a number of workshops and lectures in order to demonstrate how data science teams use analytical tools to generate business value at their company.

The impact of inviting relevant guest speakers from industry and curriculum design changes (e.g., new core modules and new elective modules) had a significant impact on sentiment for the overall programme rating for the 2018-19 and 2019-20 academic years (see Fig. 5). Using the 2016-17 programme as a baseline rate of 3.5 out of 5, sentiment increased to 4.47 in the 2019-20 academic year. While sentiment for the overall programme rating moved in a positive direction, there was a dip in sentiment in 2017-18, which can be attributed to the timing of interventions and the period of adjustment needed to have an impact on the programme.

These new module changes were informed by student feedback and used to influence staff responsible for designing and approving new modules. For example, ‘Python’, a popular programming language used in the analytics field, was strongly suggested by students from the 2017-18 cohort to be added as a new module. In response, a new module that includes Python, called ‘Advanced Programming for Business Analytics’ has since been designed and incorporated into the programme.

Although curriculum design changes will continue to be implemented, these changes are not simply to improve a superficial level of student sentiment but rather to provide students with the right content and appropriate supports that will enable them to shift from ‘surface learning’ to ‘deep learning’ of the threshold concepts related to business analytics (Ashwin, 2016). The culmination of the changes made to the curriculum design and delivery have had an overall positive impact on student sentiment and overall academic performance.

## Discussion, Recommendations, Implications and a Research Agenda

Viewing students as active participants and creators of knowledge is important because the focus of their work in the 21st century will be forging relationships, tackling novel challenges and synthesizing ‘big picture’ scenarios (Pink, 2005). This changing role of the student is akin to ‘self-authorship’ as proposed by Magolda (2004), whereby students develop the capacity to define their beliefs, values, and relationships with others. This is important as it will influence how students spend their time and how they come to see themselves as students and graduates (Brown & Knight, 1994). In addition, the wider context of student learning needs to be considered. For example, a recent study by Foltýnek and Glendinning (2015) identified that only 50 % of students in Ireland confirmed that they received training for scholarly academic writing and avoidance of plagiarism.

As there is a lack of research examining the value of LA to support curriculum design (Dyckhoff et al., 2013; Mor et al., 2015; Sergis & Sampson, 2017), there is a risk that LA is not used appropriately and thereby, its real value not realised. Our study showed that integrating LA with teacher inquiry, advantageously informed curriculum redesign. By combining LA with interviews, we actively involved students in evaluating and redesigning curriculum and therefore gained critical insight into how students experienced their learning (Bovill et al., 2011; Bovill, 2013; Trowler & Trowler, 2010).

In doing so, the student voice (cf. Campbell et al., 2007; Seale, 2009) clarified and challenged our approach to curriculum development. While we cannot assume that students will always appreciate changes in curriculum design (Brooman et al., 2015), the following inter-related recommendations are intended to support educators to realise the value of LA in the context of curriculum design, as well as to provide a more positive student learning experience. In Table 7 below, each recommendation is also mapped to the relevant assimilation stage that it relates to if implemented effectively. These recommendations are based on the case studied and synergies between the LA and curriculum design previously outlined.

**Table 7:** Mapping of recommendations to assimilation stages (X denotes application of LA)

	Initiation	Adoption	Adaptation	Acceptance	Routinisation	Infusion
Create an analytics culture	X	X	X	X	X	X
Establish baseline learning analytics		X		X		
Use learning analytics in context			X	X	X	
Create inclusive learning analytics				X	X	X
Differentiate features of sentiment data					X	X

### Create a LA Culture

Support educators to adapt, apply, and integrate LA into their teacher inquiry (Mandinach, 2012). This implies that educators will require training in the use of analytical tools and analysis of data. Tailored training is important as Vatrapu (2011) highlights that LA solutions that do not incorporate diverse “alternates for action” might not achieve the desired results for students and educators. Tailored training will address the issue of low ‘data literacy’ competency that has hindered the adoption of LA (Marsh & Farrell, 2014). From the analysis conducted in this study, this is a recommendation that if not followed, can undermine all aspects of assimilation. To even initiate the adoption process, there has to be at least some educators who believe in the value of LA, and to build an awareness of what analytics solutions exist, or what analytics features of currently used technology is not being used. At the advanced, infusion stage of assimilation, there needs to be a culture of analytics experimentation- a trial and error use of analytics, where the educators are willing to continually revise their analytics design and use, and to continually scrutinise the analytics information for any omissions or misinformation that may undermine the analytics initiative, create cynicism around it, and may damage the analytics culture in subsequent learning cycles.

### Establish Baseline Learning Analytics

Such a baseline is critically important in learning institutions where a analytics technology investment may need to be justified. Rather than adopting the technology for the sake of it, an analytics initiative can be justified by establishing baseline analytics and then showing the efficacy of the technology through a pilot on one class or module. Then, for subsequent educator and student acceptance, it is important to determine the improvement gleaned from this LA. As student feedback can be emotive, it is critical that educators establish baseline metrics from which to their build analytic capabilities, and over time, identify patterns and trends, rather than prematurely acting on negative and positive feedback. Establishing a baseline has been identified as a useful indicator for progress in other studies.

### Use Learning Analytics in Context

Understanding the contextual factors of teaching and learning is critical when determining curriculum changes, rather than purely relying on learning analytics. Of course, the most logical part of assimilation to consider context is at the adaptation stage, when such adaptations can adjust to the context of the classroom. However, traditional assimilation theory would suggest that while former adaptation decisions are certainly important, it is the fluid, minute contextual changes that technology users make that are usually the most impactful and ultimately lead to acceptance and routinisation (or not) (Gallivan et al., 2001). Therefore, we suggest that, while the strategy for implementing analytics should certainly have a formal component that makes university-wide decisions about the tailoring of the technology and its use, we would encourage the creation of an environment where educators are free to further tailor to the minutia of their module, curriculum and student context. LA should also be used to support students to develop their critical thinking and problem solving through the process of reflecting and acting on data, rather than simply a tool to generate evidence for quality assurance (cf. Tsai et al., 2019).

### Create Inclusive Learning Analytics

Educators need to design LA that will facilitate the learning of a more diverse group of learners. Apart from very obvious reasons why any initiative should be inclusive, it is also clear that such inclusivity improves both acceptance and routinisation metrics - the more educators and learners included in an initiative the higher the acceptance rate and the more that potentially use the technology in a routinised way as part of their day-to-day education activity. This implies we need to value what individual students bring to the curriculum design process (Sorenson, 2001, Bovill et al., 2011). Specifically, while inclusion in information systems has received significant attention in recent years (Coleman et al., 2017; Trauth, 2017), research on inclusion within IS curriculum design and delivery has not received sufficient attention.

### Differentiate Features of Sentiment Data

This study showed that sentiment analysis adds data points and information that adds different value to other types of information from and on students and their learning. Sentiment analysis can sense issues the students themselves may not even be aware of or know how to articulate themselves through the traditional survey. Traditional surveys are limited and subject to bias (Ochoa, 2016) in that they only elicit what the survey designer asks, and so may miss crucial issues or issues that emerge after the survey was designed. Sentiment analysis can track emerging behaviours and use of keywords in an organic and grounded manner. However, we recommend that educators consider these differences, use these instruments accordingly, and ensure they consider these differences when acting on the emerging sentiment feedback. This can be done not just to enable routinisation but to sustain it. Also, we propose that emergent analytics can then be used as a seed to initiate new and infused use of such analytics in ways that may not be obvious or indeed possible at the initial point of the technology’s adoption.

### Implications for Teacher Inquiry

We acknowledge that the recommendations provided are not exhaustive but they do however contribute to the wider discourse on the need for more academic research that provides recommendations to educators (Sergis & Sampson 2017), in order to maximise the use of LA. While this study highlights the value of actively engaging students in curriculum design (Bovill et al., 2011; Bovill, 2013; Trowler & Trowler, 2010), it should not be used to undermine the domain expertise of educators and their role in teacher inquiry.

While LA can be used to support inclusive teaching and learning, it should be used as part of a suite of tools and frameworks rather than be used in isolation. For example, the *Application of Good Practice Framework* proposed by Chickering and Gamson outlines six powerful forces in teaching: (i) activity, (ii) expectations, (iii) co-operation, (iv) interaction, (v) diversity, and (vi) responsibility. While each of the principles in the framework is in itself beneficial, when all are present they form more than the sum of their individual parts. These forces hold meaning for students from diverse backgrounds who are usually ‘under-represented’ groups, namely international students, mature students, students with (hidden/visible) disabilities, students from minority backgrounds (Ashwin et al., 2020; Larkin & Richardson, 2013). This is important because good teaching needs to provide supportive academic environments that facilitate the learning of a more diverse group of learners (Larkin & Richardson, 2013).

This study reports the positive use of student evaluations to inform teacher inquiry. It is however, important to highlight that other studies (e.g., Hornstein; 2017; Heffernan, 2021; Westoby et al., 2021) have reported the negative impact of such evaluations, whereby educators have been subject to discriminatory evaluations based on their gender, race and age, and the impact of such discrimination on their workload and mental health.

### Future Research Agenda

We acknowledge three limitations of this study, which also offer directions for future research. First, conventional textual sentiment analysis was not conducted due to limited data points, making it difficult for the predictions. Second, the findings are based on a single case which by nature, limits generalisability (cf. Yin, 2009). The findings were however based on four iterations (e.g., within-case analyses) of a one-year master’s programme and in-depth background to the case studied and rich contextual data was provided, which can help readers to tailor and apply the recommendations to their own educational context. Third, while LA has become increasingly popular, it is only one approach to inform curriculum design. It should, therefore, not be used in isolation but rather to complement other data sources (i.e., academic analytics) and the knowledge possessed by educators and curriculum designers. Based on the analysis and limitations of this study, Table 8 provides a future research agenda. It contains sample research questions associated with each recommendation that individually and collectively can improve the efficacy of LA initiatives.

**Table 8:** Research agenda

Recommendation	Example Future Research Questions
Create an analytics culture	• How can an analytics culture by implemented or enhanced in education institutions and settings? • How can the strategy for creating an analytics culture align with the general strategy of the educational institution? • How is a LA culture measured? • Does the culture need to vary according to the choice of LA tool?
Establish baseline learning analytics	• What is the most effective point in time to establish baseline analytics, given different temporal rhythms and events (e.g., start of term, start of module, date analytics first implemented, date after analytics training received)?
Use learning analytics in context	• What contextual factors affect the efficacy of LA? • How can these factors, and the impact of them on analytics use be effectively identified? • How does one balance the contextual factors that may change the nature of analytics use with the ‘textbook’ instructions of the analytics tool that suggests instructions are fully adhered to?
Create inclusive learning analytics	• How can LA technologies classify student and staff characteristics to allow effective analysis of different groups? • How can analytics analyse different degrees of diversity effectively (e.g., different levels of disability severity)? • How can analytics be used to ensure inclusivity of students who may not self-declare their specific differ?
Differentiate features of sentiment data	• How can sentiment analysis cater for class variety (e.g., large vs. small class, undergraduate vs. postgraduate or Ph.D., single domain or multi-discipline)? • How can sentiment analysis be calibrated to ensure privacy and potentially sensitive issues and correlations are not included?

## Conclusions

Learning analytics is a research field that aims to support educators during the process of inquiry. This study reported the value of using sentiment analytics as a form of LA to improve student-learning experiences and inform curriculum design. Sentiment analytics offers a dynamic and evidence-based approach to guide teacher inquiry and inform curriculum design. However, it assumes that educators have the ability to use these types of analytical tools and techniques and align these with their teacher inquiry. This is most likely not the case in many universities, due to a range of factors including, (i) the capacity of the discipline, (ii) availability of funding, (iii) tailored training in the use of LA, and (iv) continuous support in the use of LA and curriculum design.

## Appendix

**Table 9:** Module & programme feedback questions

Module		
Q	Question	Type
1	The expected learning outcomes of the module are clear to me. (Note: the learning outcomes are included in the module outline if you wish to review them)	Opinion Scale/Likert
2	The coursework, learning activities and assessment match the learning outcomes (e.g., prescribed readings, problem-solving, discussions, tutorials, workshops).	Opinion Scale/Likert
3	The module materials support my learning (e.g., handouts, slides, Blackboard, references, readings).	Opinion Scale/Likert
4	The module is well organised.	Opinion Scale/Likert
5	The teaching staff explain difficult concepts and topics effectively.	
6	The lectures inspire me to want to learn more.	Opinion Scale/Likert
7	Please comment on your response to this question.	Open-ended /narrative
8	What do you like about this module?	Open-ended /narrative
9	What do you dislike about this module?	Open-ended /narrative
10	What suggestions can you offer that would help make this module a more valuable learning experience for you?	Open-ended /narrative
11	Please rate your personal overall satisfaction with this module as a learning experience.	Opinion Scale/Likert
Programme Feedback		
Q	Question	Type
1	The learning outcomes of the programme were clearly communicated to me.	Opinion Scale/Likert
2	The programme delivered the expected learning outcomes	Opinion Scale/Likert
3	The assessment and examination requirements were clearly communicated to me	Opinion Scale/Likert
4	The modules on the programme were linked effectively	Opinion Scale/Likert
5	If you were to remove one module, it would be? Please explain you reason for removing this module.	Open-ended /narrative
6	If you could add a new topic, it would be? Please explain why this topic would add value to the programme.	Open-ended /narrative
7	I found the programme intellectually stimulating	Opinion Scale/Likert
8	I received helpful and timely feedback during the programme	Opinion Scale/Likert
9	The teaching methods were effective for this type of programme	Opinion Scale/Likert
10	One way to improve the teaching methods would be…	Open-ended /narrative
11	The library and IT facilities met my requirements	Opinion Scale/Likert
12	The professional experience activities associated with the programme were satisfactory.	Opinion Scale/Likert
13	How much do you think you have gained from studying this programme?	Opinion Scale/Likert
14	Finally, please make some suggestions of ways in which this programme might be improved	Open-ended /narrative

## References

[CR1] Ahmad M-O, Dennehy D, Conboy K, Oivo M (2018). Kanban in software engineering: A systematic mapping study. Journal of Systems and Software.

[CR2] Ashwin A (2016). *Assessing Threshold Concepts and Learning in Economics and Business*.

[CR3] Ashwin, P., Boud, D., Calkins, S., Coate, K., Hallett, F., Light, G., McArthur, J., MacLaren, I., McCune, V. (2020). *Reflective teaching in Higher Education*. Bloomsbury Academic

[CR4] Avramides K, Hunter J, Oliver M, Luckin R (2015). A method for teacher inquiry in cross-curricular projects: Lessons from a case study. British Journal of Educational Technology.

[CR5] Bach, C. (2010). Learning analytics: Targeting instruction, curricula and student support. Office of the Provost, Drexel University. http://www.iiis.org/CDs2010/CD2010SCI/EISTA_2010/PapersPdf/EA655ES.pdf. Accsessed 4 Mar 2021

[CR6] Bakharia, A., Corrin, L., De Barba, P., Kennedy, G., Gašević, D., Mulder, R. … Lockyer, L. (2016). A conceptual framework linking learning design with learning analytics. In: *Proceedings of the Sixth International Conference on Learning Analytics & Knowledge* (p. 329–338). ACM.

[CR7] Baxter-Magolda MB (2003). Identity and learning: Student affairs’ role in transforming higher education. Journal of College Student Development.

[CR8] Bharati P, Chaudhury A (2019). Assimilation of big data innovation: Investigating the roles of IT, social media, and relational capital. Information Systems Frontiers.

[CR9] Biggs J (1996). Enhancing teaching through constructive alignment. Higher Education.

[CR10] Biggs J (1999). What the student does: Teaching for enhanced learning. Higher Education Research & Development.

[CR11] Biggs, J. (2003). *Aligning teaching for constructing learning *(pp. 1–4). Higher Education Academy

[CR12] Bos, N., & Brand-Gruwel, S. (2016). Student differences in regulation strategies and their use of learning resources: Implications for educational design. In: *Proceedings of the Sixth International Conference on Learning Analytics & Knowledge* (p. 344–353). ACM

[CR13] Bovill C (2013). An investigation of co-created curricula within higher education in the UK, Ireland and the USA. Innovations in Education and Teaching International.

[CR14] Bovill C, Cook-Sather A, Felten P (2011). Students as co‐creators of teaching approaches, course design, and curricula: implications for academic developers. International Journal for Academic Development.

[CR15] Brooman S, Darwent S, Pimor A (2015). The student voice in higher education curriculum design: is there value in listening?. Innovations in Education and Teaching International.

[CR16] Brown S, Knight P (1994). Assessing learners in higher education.

[CR17] Campbell, F., Beasley, L., Eland, J., & Rumpus, A. (2007). *Hearing the student voice: Final report*. HEA, Subject Centre for Education, Napier University. Retrieved from http://dera.ioe.ac.uk/13053/2/3911.pdf. Accessed 15 Feb 2021

[CR18] Cao Y, Ajjan H, Hong P, Le T (2018). Using social media for competitive business outcomes: an empirical study of companies in China. Journal of Advances in Management Research.

[CR19] Chatterjee S, Rana NP, Dwivedi YK (2021). How does business analytics contribute to organisational performance and business value? A resource-based view.

[CR20] Clow D (2013). An overview of learning analytics. Teaching in Higher Education.

[CR21] Coleman, E., Carter, M., Davison, R. M., Chigona, W., & Urquhart, C. (2017) Social Inclusion in the AIS Community: What, Why and How?. ICIS 2017 Proceedings. 4. http://aisel.aisnet.org/icis2017/Panels/Presentations/4. Accessed 4 Mar 2021

[CR22] Conole, G. (2012). *Designing for learning in an open world* (Vol. 4). Springer

[CR23] Cooper RB, Zmud RW (1990). Information technology implementation research: a technological diffusion approach. Management Science.

[CR24] Cousin G (2006). An introduction to threshold concepts. Planet.

[CR25] Dana N, Yendol-Hoppey D (2014). The reflective educator’s guide to classroom research: Learning to teach and teaching to learn through practitioner inquiry.

[CR26] Dennehy, D. (2020). Ireland After the Pandemic: Utilising AI to Kick-Start a Sustainable Economic Recovery. *Cutter Business Technology Journal*

[CR27] Dunbar RL, Dingel MJ, Prat-Resina X (2014). Connecting analytics and curriculum design: process and outcomes of building a tool to browse data relevant to course designers. Journal of Learning Analytics.

[CR28] Dyckhoff, A. L., Lukarov, V., Muslim, A., Chatti, M. A., & Schroeder, U. (2013). Supporting action research with learning analytics. In: *Proceedings of the Third International Conference on Learning Analytics and Knowledge* (p. 220–229). ACM

[CR29] Feldman A, Altrichter H, Posch P, Somekh B (2018). Teachers investigate their work: An introduction to action research across the professions.

[CR30] Ferguson R (2012). Learning analytics: drivers, developments and challenges. International Journal of Technology Enhanced Learning.

[CR31] Fichman RG, Zmud RW (2000). The diffusion and assimilation of information technology innovations. Framing the domains of IT research: Glimpsing the future through the past.

[CR32] Fichman RG, Kemerer CF (1997). The assimilation of software process innovations: An organizational learning perspective. Management Science.

[CR33] Fielding M (2001). Students as radical agents of change. Journal of Educational Change.

[CR34] Foltýnek T, Glendinning I (2015). Impact of policies for plagiarism in higher education across Europe: Results of the project. Acta Universitatis Agriculturae et Silviculturae Mendelianae Brunensis.

[CR35] Gallivan MJ (2001). Organizational adoption and assimilation of complex technological innovations: development and application of a new framework. ACM SIGMIS Database.

[CR36] Gasevic D, Dawson S, Rogers T, Gasevic D (2016). Learning analytics should not promote one size fits all: The effects of instructional conditions in predicating academic success.

[CR37] Gašević D, Kovanović V, Joksimović S (2017). Piecing the learning analytics puzzle: A consolidated model of a field of research and practice. Learning: Research and Practice.

[CR38] Gordon SL (2017). The sociology of sentiments and emotion. Social Psychology.

[CR39] Greller W, Ebner M, Schön M, Kalz M, Marco R (2014). Learning analytics: From theory to practice–data support for learning and teaching. Computer assisted assessment. Research into E-Assessment.

[CR40] Gupta S, Drave VA, Dwivedi YK, Baabdullah AM, Ismagilova E (2020). Achieving superior organizational performance via big data predictive analytics: A dynamic capability view. Industrial Marketing Management.

[CR41] Hansen C, Wasson B (2016). Teacher inquiry into student learning:-The TISL heart model and method for use in teachers’ professional development. Nordic Journal of Digital Literacy.

[CR42] Hattie J (2008). Visible learning: A synthesis of over 800 meta-analyses relating to achievement.

[CR43] Heffernan, T. (2021). Sexism, racism, prejudice, and bias: a literature review and synthesis of research surrounding student evaluations of courses and teaching. *Assessment & Evaluation in Higher Education* (pp. 1–11)

[CR44] Henritius E, Löfström E, Hannula MS (2019). University students’ emotions in virtual learning: a review of empirical research in the 21st century. British Journal of Educational Technology.

[CR45] Heritage M, Kim J, Vendlinski T, Herman J (2009). From evidence to action: A seamless process in formative assessment?. Educational Measurement: Issues and Practice.

[CR46] Hernández-Leo D, Martinez‐Maldonado R, Pardo A, Rodríguez‐Triana MJ (2019). Analytics for learning design: A layered framework and tools. British Journal of Educational Technology.

[CR47] Hornstein HA (2017). Student evaluations of teaching are an inadequate assessment tool for evaluating faculty performance. Cogent Education.

[CR48] Hounsell, D., & Hounsell, J. (2007). Teachinglearning environments in contemporary mass higher education. In *BJEP Monograph Series II, Number 4 - Student Learning and University Teaching* (pp. 91–111). British Psychological Society

[CR49] Jongeling, R., Datta, S., & Serebrenik, A. (2015). Choosing your weapons: On sentiment analysis tools for software engineering research. In* Proceedings of the 2015 IEEE International Conference on Software Maintenance and Evolution (ICSME) *(pp. 531–535). 10.1109/ICSM.2015.7332508

[CR50] Knight S, Gibson A, Shibani A (2020). Implementing learning analytics for learning impact: Taking tools to task. The Internet and Higher Education.

[CR51] Land R, Neve H, Martindale L (2018). Threshold concepts, action poetry and the health professions: An interview with Ray Land. International Journal of Practice-based Learning in Health and Social Care.

[CR52] Larkin H, Richardson B (2013). Creating high challenge/high support academic environments through constructive alignment: student outcomes. Teaching in Higher Education.

[CR53] Liang H, Saraf N, Hu Q, Xue Y (2007). Assimilation of Enterprise systems: The effect of institutional pressures and the mediating role of top management. MIS Quarterly.

[CR54] Linnenbrink-Garcia L, Pekrun R (2011). Students’ emotions and academic engagement: Introduction to the special issue. Contemporary Educational Psychology.

[CR55] Liu B (2010). Sentiment analysis and opinion mining. Synthesis Lectures on Human Language Technologies.

[CR56] Lockyer L, Heathcote E, Dawson S (2013). Informing pedagogical action: Aligning learning analytics with learning design. American Behavioral Scientist.

[CR57] Magolda, B. (2004). Self-authorship as the common goal. *Learning partnerships: Theory and models of practice to educate for self-authorship* (pp. 1–35)

[CR58] Mainhard T, Oudman S, Hornstra L, Bosker RJ, Goetz T (2018). Student emotions in class: The relative importance of teachers and their interpersonal relations with students. Learning and Instruction.

[CR59] Mandinach E (2012). A perfect time for data use: Using data driven decision making to inform practice. Educational Psychologist.

[CR60] Mangaroska K, Giannakos M (2018). Learning analytics for learning design: A systematic literature review of analytics-driven design to enhance learning. IEEE Transactions on Learning Technologies.

[CR61] Marsh, J-A., & Farrell, C-C. (2014). How leaders can support teachers with data-driven decision making A framework for understanding capacity building. *Educational Management Administration & Leadership* (pp. 1–21)

[CR62] Mayer-Schönberger V, Cukier K (2013). Big data: A revolution that will transform how we live, work, and think.

[CR63] Means B, Chen E, DeBarger A (2011). Teachers’ ability to use data to inform instruction: challenges and supports.

[CR64] Mega C, Ronconi L, De Beni R (2014). What makes a good student? How emotions, self-regulated learning, and motivation contribute to academic achievement. Journal of Educational Psychology.

[CR65] Meyer AD, Goes JB (1988). Organizational assimilation of innovations: A multilevel contextual analysis. Academy of Management Journal.

[CR66] Meyer JH, Land R (2005). Threshold concepts and troublesome knowledge (2): Epistemological considerations and a conceptual framework for teaching and learning. Higher Education.

[CR67] Mor Y, Ferguson R, Wasson B (2015). Learning design, teacher inquiry into student learning and learning analytics: A call for action. British Journal of Educational Technology.

[CR68] Nicola-Richmond K, Pépin G, Larkin H, Taylor C (2018). Threshold concepts in higher education: A synthesis of the literature relating to measurement of threshold crossing. Higher Education Research & Development.

[CR69] Oblinger DG (2012). Let’s Talk… Analytics. EDUCAUSE Review.

[CR70] Ochoa, X. (2016). Simple metrics for curricular analytics. In *Proceedings of the 1st Learning Analytics for Curriculum and Program Quality Improvement Workshop*. Edinburgh, United Kingdom, p. 20–26.

[CR71] Olah L, Lawrence N, Riggan M (2010). Learning to learn from benchmark assessment data: How teachers analyze results. Peabody Journal of Education.

[CR72] Ooi KB, Lee VH, Tan GWH, Hew TS, Hew JJ (2018). Cloud computing in manufacturing:the next industrial revolution in Malaysia?. Expert Systems with Applications.

[CR73] Orlikowski WJ (1993). CASE tools as organizational change: Investigating incremental and radical changes in systems development. MIS Quarterly.

[CR74] Papamitsiou Z, Economides A (2014). Learning analytics and educational data mining in practice: A systematic literature review of empirical evidence. Educational Technology & Society.

[CR75] Pekrun R, Goetz T, Titz W, Perry RP (2002). Academic emotions in students’ self-regulated learning and achievement: A program of qualitative and quantitative research. Educational psychologist.

[CR76] Picciano AG (2012). The evolution of big data and learning analytics in American Higher Education. Journal of Asynchronous Learn Network.

[CR77] Pink DH (2005). A whole new mind: Moving from the information age to the conceptual age.

[CR78] Roden S, Nucciarelli A, Li F, Graham G (2017). Big data and the transformation of operations models: a framework and a new research agenda. Production Planning & Control.

[CR79] Rudduck J, Thiessen D, Cook-Sather A (2007). Student voice, student engagement, and school reform. International Handbook of Student Experience in Elementary and Secondary School.

[CR80] Saraf N, Liang H, Xue Y, Hu Q (2013). How does organisational absorptive capacity matter in the assimilation of enterprise information systems?. Information Systems Journal.

[CR81] Seale J (2009). Doing student voice work in higher education: An exploration of the value of participatory methods. British Educational Research Journal.

[CR82] Sergis, S., & Sampson, D. G. (2017). Teaching and learning analytics to support teacher inquiry: A systematic literature review. *In (Ed.), Learning analytics: Fundaments, applications, and trends* (pp. 25–63). Springer

[CR83] Siemens, G. (2012, April). Learning analytics: envisioning a research discipline and a domain of practice. In: *Proceedings of the 2nd international conference on learning analytics and knowledge* (pp. 4–8). ACM

[CR84] Siemens G, Long P (2011). Penetrating the fog: Analytics in learning and education. EDUCAUSE Review.

[CR85] Sorenson L, Miller JE, Groccia JE, Miller MS (2001). College teachers and student consultants: Collaborating about teaching and learning. Student-assisted teaching.

[CR86] Stake, R. E. (2000). Case studies. In N. K. Denzin & Y. S. Lincoln (Eds.), *Handbook of Qualitative Research* (pp. 435-453). Sage

[CR87] Tausczik YR, Pennebaker JW (2010). The psychological meaning of words: LIWC and computerized text analysis methods. Journal of Language and Social Psychology.

[CR88] Trauth E (2017). A research agenda for social inclusion in information systems. ACM SIGMIS Database: The Database for Advances in Information Systems.

[CR89] Trowler, V., & Trowler, P. (2010). Student engagement evidence summary. Higher Education Academy, *11*(1), 1–15.

[CR90] Tsai YS, Poquet O, Gašević D, Dawson S, Pardo A (2019). Complexity leadership in learning analytics: Drivers, challenges and opportunities. British Journal of Educational Technology.

[CR91] Van Barneveld A, Arnold KE, Campbell JP (2012). Analytics in higher education: Establishing a common language. EDUCAUSE Learning Initiative.

[CR92] Vatrapu, R. (2011). Cultural considerations in learning analytics. In P. Long, G. Siemens, G. Conole, & D. Gasevic (Eds.), *Proceedings of the 1st International Conference on Learning Analytics and Knowledge* (pp. 127–133). Association for Computing Machinery

[CR93] Wamba SF, Akter S, Edwards A, Chopin G, Gnanzou D (2015). How ‘big data’can make big impact: Findings from a systematic review and a longitudinal case study. International Journal of Production Economics.

[CR94] Wang X, Conboy K, Pikkarainen M (2012). Assimilation of agile practices in use. Information Systems Journal.

[CR95] Weibl, J., & Hess, T. (2018). Success or failure of big data: Insights of managerial challenges from a technology assimilation perspective. *Proceedings of the Multikonferenz Wirtschaftsinformatik (MKWI)*, (pp. 12–59)

[CR96] Westoby, C., Dyson, J., Cowdell, F., & Buescher, T. (2021). What are the barriers and facilitators to success for female academics in UK HEIs? A narrative review. *Gender and Education *(pp*. *1–24)

[CR97] Wilson, T., Wiebe, J., & Hoffmann, P. (2005). Recognizing contextual polarity in phrase-level sentiment analysis. In* Proceedings of the Human Language Technology and Empirical Methods in Natural Language Processing *(pp. 347–354)*.* Association for Computational Linguistics

[CR98] Timperley H, Wilson, A., Barrar, H., & Fung, I. (2010). Teacher Professional Learning and Development. Report for the New Zealand Ministry of Education. http://www.oecd.org/edu/school/48727127.pdf. Accessed 4 Mar 2021

[CR99] Yin RK (2009). Case study research: Design and methods.

[CR100] Young V, Kim D (2010). Using assessments for instructional improvement: A literature review. Education Policy Analysis Archives.

[CR101] Zhang L, Wang S, Liu B (2018). Deep learning for sentiment analysis: A survey. Wiley Interdisciplinary Reviews: Data Mining and Knowledge Discovery.

[CR102] Zhu K, Kraemer KL, Xu S (2006). The process of innovation assimilation by firms in different countries: a technology diffusion perspective on e-business. Management Science.

[CR103] Zhu ZT, Yu MH, Riezebos P (2016). A research framework of smart education. Smart Learning Environments.

